# Complete mitochondrial genome of the six-line wrasse *Pseudocheilinus hexataenia* (Labriformes, Labridae)

**DOI:** 10.1080/23802359.2021.2017367

**Published:** 2022-01-05

**Authors:** Sang-Eun Nam, Hye-Jin Eom, Hyoung Sook Park, Jae-Sung Rhee

**Affiliations:** a Department of Marine Science, College of Natural Sciences, Incheon National University, Incheon, South Korea; b Department of Song-Do Bio-Environmental Engineering, Incheon Jaeneung University, Incheon, South Korea; c Research Institute of Basic Sciences, Incheon National University, Incheon, South Korea; d Yellow Sea Research Institute, Incheon, South Korea

**Keywords:** Complete mitogenome, labrid fish, *Pseudocheilinus hexataenia*, phylogenetic analysis

## Abstract

Here, we report the complete mitogenome information of the six-line wrasse *Pseudocheilinus hexataenia* (Bleeker, 1857). Genome sequencing using the Illumina HiSeq platform allowed the assembly of a circular mitochondrial genome of 17,111 bp from *P. hexataenia,* consisting of 54% AT nucleotides, 13 protein-coding genes (PCGs), two ribosomal RNA (rRNA) genes, 22 transfer RNA (tRNA) genes, and a putative control region in the typical Labriformes gene composition. The gene order of the *P. hexataenia* mitochondrion was identical to that of the Labridae mitogenomes. Phylogenetic reconstruction places *P. hexataenia* with a close relationship with the mitogenome of the goldsinny wrasse, *Ctenolabrus rupestris*.

The family Labridae, one of the most conspicuous polyphyletic marine fish, has over 630 species in 88 genera (Nelson et al. 2016) and shows high species richness, abundance, and structural and ecological diversity, resulting in one of the top five families in coral reefs (Bellwood 2019). Labrids include a large group of both wrasses and parrotfish, crucial in many coral reef ecosystems. In recent decades, significant progress has been made in resolving the phylogenetic relationships among the complex lineages of both tropical and temperate labrids (Westneat and Alfaro 2005; Cowman et al. 2009; Price et al. 2011; Phillips et al. 2016; Baliga and Law 2016). The six-line wrasse *Pseudocheilinus hexataenia* (Bleeker 1857) is widely distributed in the tropical marine waters of the Indo-Pacific. The six-line wrasse is a popular ornamental species because of its small size (∼10 cm), high activity in aquaria, and unique body color, recognized by the six distinct horizontal orange stripes along the violet flanks. In general, cleaner wrasses play a fundamental ecological role in interspecific symbiosis by removing parasites and infected tissues from the surfaces of fish clients. The six-line wrasse is a facultative cleaner, as it may occasionally clean, primarily at the juvenile stage. Although many whole mitogenomes have been published in the Labridae, there is no information on the complete mitogenome in Pseudocheilines. Therefore, the objective of this report is to bring together the phylogenetic information on the genus *Pseudocheilinus* as a basis for further evaluation of Labridae evolution.

A specimen of *P. hexataenia* was collected from the East China Sea (30°45 N 126°22'E) in July 2013. The specimen and DNA were deposited at the Research Institute of Basic Sciences of Incheon National University (Specimen ID: 2013- Labridae-01; https://www.inu.ac.kr/user/indexMain.do?siteId=ribs; Sang-Eun Nam; se_nam2@inu.ac.kr) (Nam and Rhee 2020). All animal handling and experimental procedures were approved by the Animal Welfare Ethical Committee and the Animal Experimental Ethics Committee of the Incheon National University (Incheon, South Korea). Genomic DNA was prepared from a specimen muscle using a DNeasy Blood and Tissue kit (Qiagen, Hilden, Germany). A fragment library was prepared using the TruSeq DNA Sample Preparation Kit (Illumina, San Diego, CA, USA). The sequencing library was prepared by random fragmentation of the DNA sample, followed by 5′ and 3′ adapter ligation. Raw reads were obtained from the sample that passed the quality control check on the Illumina HiSeq platform (Illumina) at Macrogen, Inc. (Seoul, South Korea). Adapter sequences, low-quality reads, reads with > 10% of unknown bases, and ambiguous bases were removed from FASTAQ with Trimmomatic ver. 0.40 (Illumina) using default trimming steps. After the quality check process, 35,698,204 filtered reads were obtained from 45,836,020 raw reads. Subsequently, *de novo* assembly was conducted with various k-mers using SPAdes ver. 3.12.0 (Bankevich et al. 2012) with default settings and the “isolate” option enabled. The resulting circular contig consensus sequence was annotated using MITOS2 (Bernt et al. 2013) and tRNAscan-SE 2.0 (Lowe and Eddy 1997). BLAST searches confirmed the identity of the genes (http://blast.ncbi.nlm.nih.gov).

The *P. hexataenia* circular 17,111 bp mitogenome (GenBank accession no. MZ357706) was composed of the following nucleotide composition: 27.3% A, 29.0% C, 17.6% G, and 26.2% T. The gene order and composition of the *P. hexataenia* mitogenome are identical to those of all known wrasse mitogenomes. Thirteen *P. hexataenia* mitochondrial PCGs begin with an ATG start codon, and the 22 tRNAs have typical cloverleaf secondary structures. We reconstructed a phylogenetic tree using the concatenated set of all 13 PCGs of the *P. hexataenia* mitogenome, 24 published complete mitogenomes belonging to Labriformes, and two Centrarchiformes species as an outgroup (Figure 1). JModelTest ver. 2.1.10 (Darriba et al. 2012) was used to select the best substitution model, and a substitution model (HKY + G + I) was applied to perform a maximum-likelihood (ML) method in PhyML ver. 2.4.5 (Guindon and Gascuel 2003) with 1000 bootstrap replicates. The *P. hexataenia* mitogenome formed a sister group with the mitogenome of the goldsinny wrasse, *C. rupestris*, with strong support. The overall topology was consistent with previous phylogenetic results (Phillips et al. 2016). Our results also agree that Pseudocheilines, including *Pseudocheilinus*, were not the sister-group to the Cheilines and were split off from the Cheilines and placed at the next node up the tree (Westneat and Alfaro 2005). Additional whole mitogenomes of Pseudocheilines and Labrines are needed for a more detailed analysis of their phylogenetic relationships.

**Figure 1.**
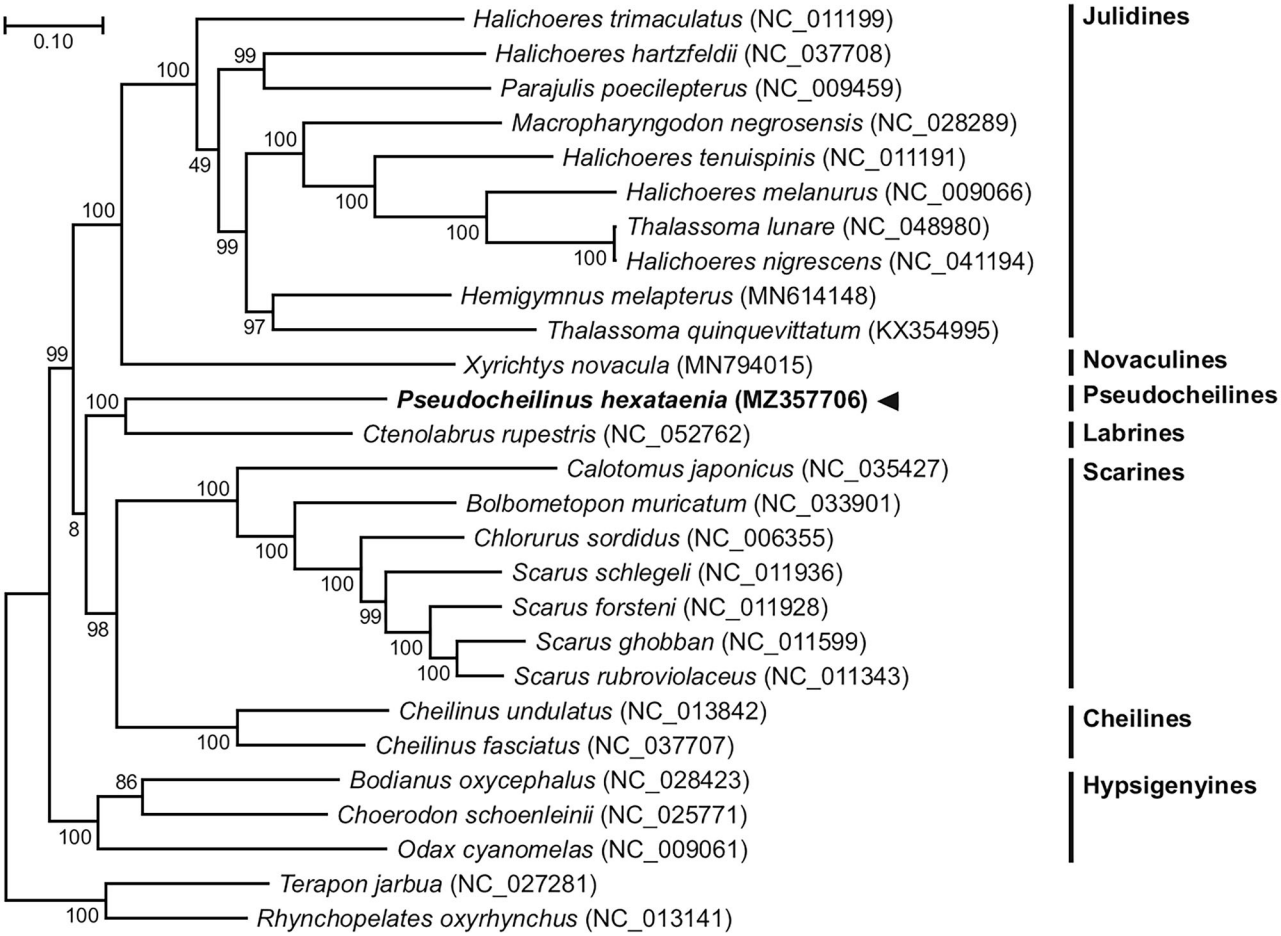
Maximum-likelihood (ML) phylogeny of 24 published complete mitogenomes belonging to Labriformes and two Centrarchiformes species as an outgroup based on the concatenated nucleotide sequences of protein-coding genes (PCGs). The phylogenetic analysis was performed using the maximum likelihood method, GTR + G + I model with a bootstrap of 1000 replicates. Numbers on the branches indicate ML bootstrap percentages. DDBJ/EMBL/Genbank accession numbers for published sequences are incorporated. The black triangle indicates the fish analyzed in this study.

## Author’ contributions

S.-E. Nam: Conceptualization, Methodology, Software, Writing

H.-J. Eom: Methodology, Software, Data curation

H.S. Park: Conceptualization, Visualization, Reviewing and Editing

J.-S. Rhee: Conceptualization, Supervision, Reviewing and Editing

## Data Availability

BioProject, BioSample, and SRA accession numbers are https://www.ncbi.nlm.ni h.gov/bioproject/PRJNA743750, https://www.ncbi.nlm.nih.gov/biosample/SAMN20059971, and https://www.ncbi.nlm.nih.gov/sra/?term=SRR15348071, respectively. The data that support the findings of this study are available at the National Center for Biotechnology Information (NCBI) at https://www.ncbi.nlm.nih.gov, with the accession number MZ357706.
